# Phlegmonous gastritis: a case series

**DOI:** 10.1186/s13256-021-02999-9

**Published:** 2021-09-06

**Authors:** Yoshikazu Yakami, Toshihiko Yagyu, Tomoki Bando

**Affiliations:** Department of Internal Medicine, Higashi-Osaka Hospital, 7-22, Chuo 1 chome, Joto-ku, Osaka, 536-0005 Japan

**Keywords:** Phlegmonous gastritis, Scirrhous gastric cancer, Gastric syphilis, Acute gastric mucosal lesion

## Abstract

**Background:**

Phlegmonous gastritis is a rare and fatal infectious disease of the stomach, presenting varied and nonspecific endoscopic images, which are therefore difficult to diagnose. This report discusses three cases of phlegmonous gastritis, each with unique endoscopic images, and considers the differential diagnosis of this disease. These cases were initially suspected of scirrhous gastric cancer, gastric syphilis, and acute gastric mucosal lesion.

**Case presentation:**

*Case 1* A 32-year-old Asian man visited our hospital complaining of upper abdominal pain. Endoscopy raised suspicion of scirrhous gastric cancer. However, a histopathological examination showed no malignant cells, thus leading to the diagnosis of phlegmonous gastritis. The patient was started on antibiotic therapy, which was effective.

*Case 2* A 33-year-old Asian man visited our hospital complaining of epigastralgia. Endoscopy raised suspicion of gastric syphilis. However, the serum test for syphilis was negative, and *Streptococcus viridans* was detected in the biopsy specimen culture, which led to the diagnosis of phlegmonous gastritis.

The patient was started on antibiotic therapy, resulting in significant improvement in the endoscopic image after 2 weeks.

*Case 3* A 19-year-old Asian man visited our hospital complaining of epigastric pain. Endoscopy raised suspicion of acute gastric mucosal lesion. A gastric juice culture showed *Pseudomonas aeruginosa* and *Streptococcus viridans*, thus leading to the diagnosis of phlegmonous gastritis. The patient was started on antibiotic therapy, resulting in the disappearance of the gastric lesions.

**Conclusion:**

In severe cases of phlegmonous gastritis, immediate surgical treatment is generally required. However, the endoscopic images are varied and nonspecific. These three cases suggest that clinicians need to consider the differential diagnosis of phlegmonous gastritis and make accurate diagnoses at an early stage.

## Background

Phlegmonous gastritis is a rare and deadly infectious disease of the gastric wall, mainly occurring in the submucosa of the stomach. This disease is caused by suppurative bacterial infection and is conventionally managed by conservative treatment using antibiotics. In severe cases, such as perforation, urgent surgical treatment is required. Certainly, its mortality rate is not low. Therefore, this disease must be diagnosed correctly at an early stage. However, considering that the endoscopic images of phlegmonous gastritis are varied and nonspecific, making an early diagnosis is difficult. Herein, we report three cases of phlegmonous gastritis, each with unique endoscopic images, and consider a differential diagnosis. These cases were initially suspected of scirrhous gastric cancer, gastric syphilis, and acute gastric mucosal lesion. In severe cases, immediate surgical treatment is generally required. Therefore, accurate diagnoses and proper treatment need to be provided.

## Case presentation

### Case 1

A 32-year-old Asian man visited our hospital, with complaints of epigastralgia, slight fever, and vomiting. He had a history of alcohol consumption (40 g/day) but had no medical history. His temperature was 37.4 °C. His white blood cell (WBC) count was 22,600/mm^3^, while his C-reactive protein level was 3.00 mg/dl. Abdominal computed tomography (CT) detected a thickened, edematous gastric wall.

**Fig. 1:**
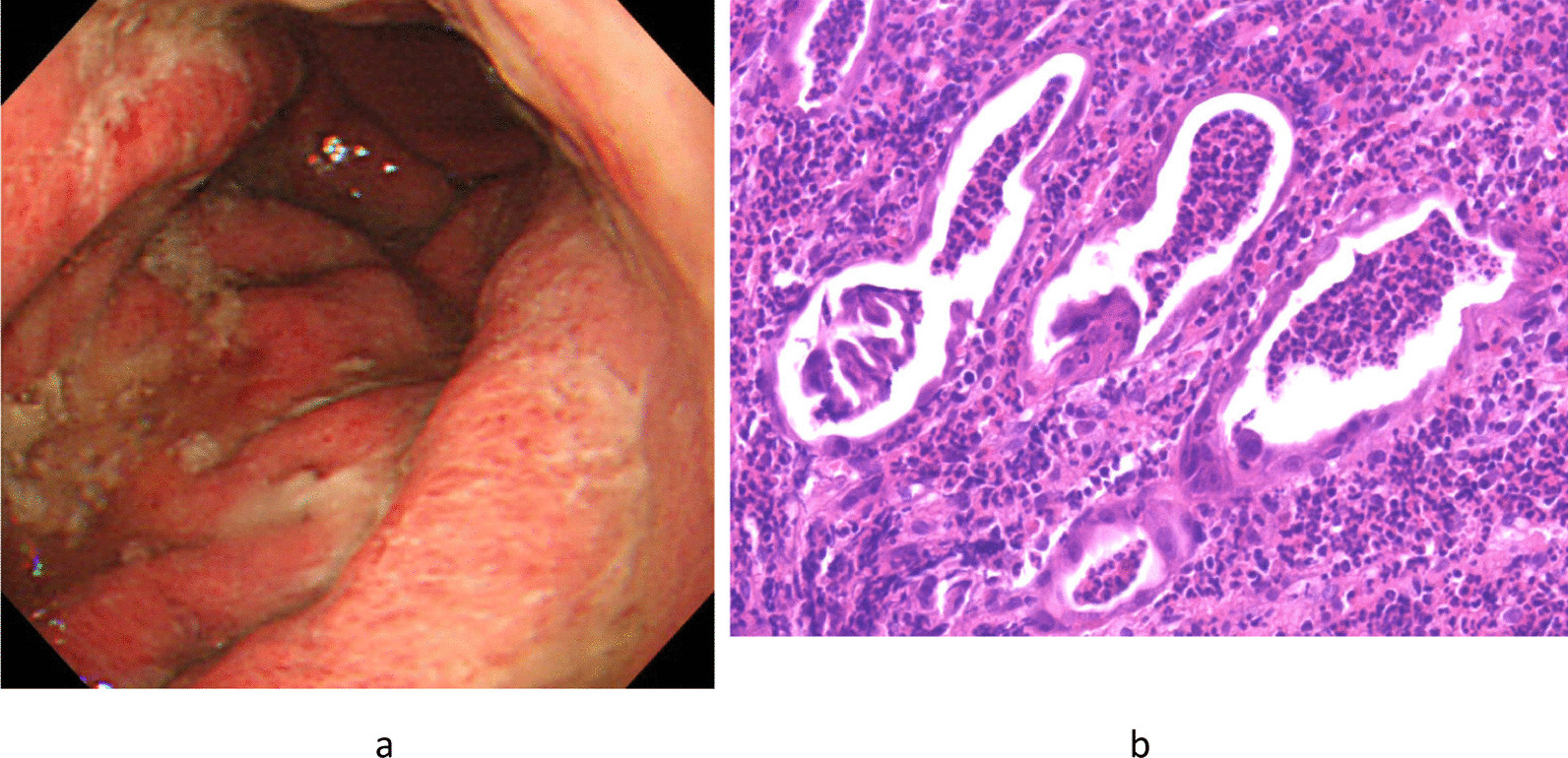
**a** Endoscopic image of case 1. Significant hyperplasia of the gastric folds with abundant mucopus, and extensive redness and edema of the gastric mucosa were observed. **b** Histological image of case 1. Mucosal necrosis and neutrophil infiltration were detected, but no malignant cells were found

Hence, the patient underwent esophagogastroduodenoscopy, which revealed a significant hyperplasia of the gastric folds with abundant mucopus, extensive redness and edema of the gastric mucosa, and poor distensibility of the gastric wall (Fig. 1a). Considering this endoscopic image, scirrhous gastric cancer was suspected. On histopathological examination, mucosal necrosis and severe neutrophil infiltration were detected, with no malignant cells (Fig. 1b).

The clinical course (increased fever, epigastric pain, and elevated WBC count) as well as imaging (gastric wall thickness on CT and endoscopic findings) and histopathological findings led us to consider the serious infection and to diagnose the patient with phlegmonous gastritis, despite the biopsy specimen culture being negative.

Antibiotic therapy (levofloxacin, 500 mg/day × 7 days) was then provided, leading to the patient’s recovery.

### Case 2

A 33-year-old Asian man with epigastralgia visited our hospital. He had a history of excessive alcohol consumption (the exact amount was unknown), had no medical history, and was sexually active. The WBC count was 12,400/mm^3^. Abdominal CT detected localized thickening of the gastric wall, mainly in the antrum.

**Fig. 2:**
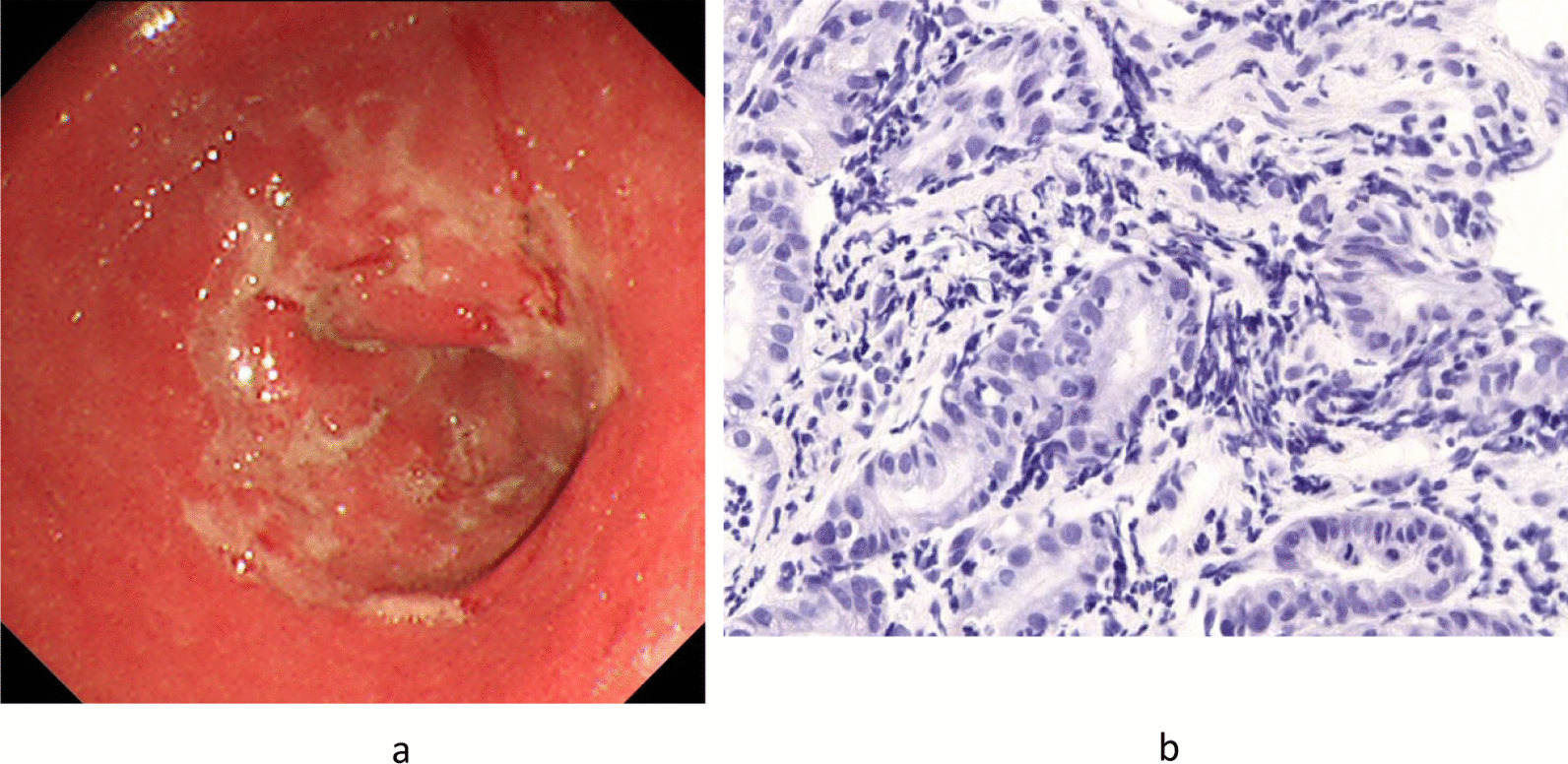
**a** Endoscopic image of case 2. Multiple shallow ulcers with slough fused into a map-like pattern were observed in the antrum. **b** Histological image of case 2. Immunostaining of the biopsy specimen was negative (*Treponema pallidum* was not found)

The endoscopic image revealed multiple shallow ulcers with slough fused into a map-like pattern in the same area (Fig. 2a).

Given the endoscopic findings, the patient was diagnosed with gastric syphilis. However, his serum test result for syphilis was negative, and the culture of biopsy specimen revealed the presence of *Streptococcus viridans*, resulting in the diagnosis of phlegmonous gastritis. In addition, immunostaining of the biopsy specimen was negative (*Treponema pallidum* was not found) (Fig. 2b). Thus, antibiotic therapy (amoxicillin, 750 mg/day × 14 days) was started. After 2 weeks, the endoscopic image revealed a remarkable improvement.

### Case 3

A 19-year-old Asian male college student visited our hospital, complaining of epigastralgia and vomiting. He had no history of drug and alcohol consumption and had no medical history. However, he had a fever (38.0 °C), with a WBC count of 15,900/mm^3^. Abdominal CT detected a thickened gastric wall, particularly in the antrum.

**Fig. 3:**
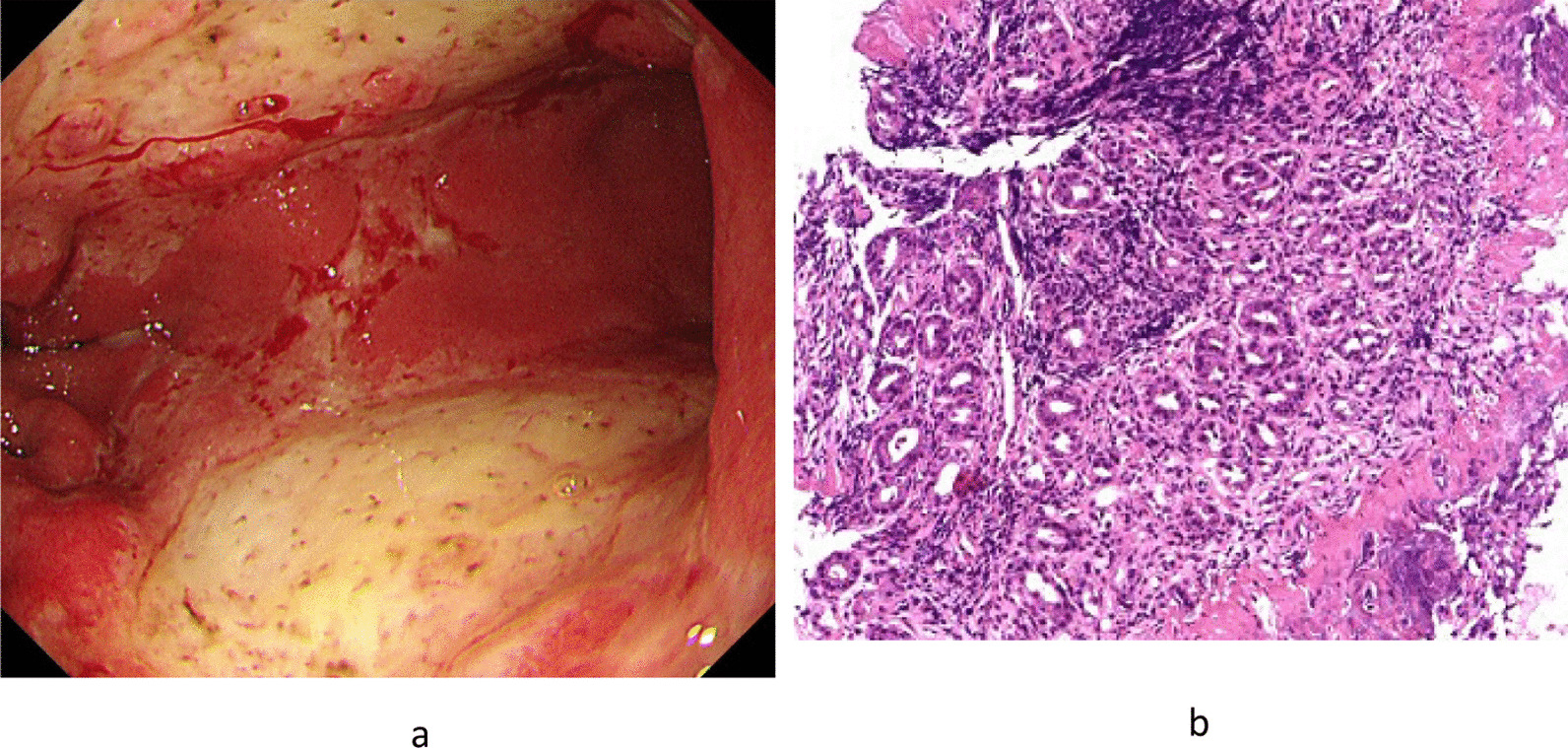
**a** Endoscopic image of case 3. Large and shallow ulcers with reddish and edematous mucosa were observed in the antrum, with some coagulation. **b** Histological image of case 3. Direct microscopy found no *Helicobacter pylori*

Endoscopic image showed large and shallow ulcers with reddish and edematous mucosa in the antrum, with some coagulation (Fig. 3a). We initially suspected acute gastric mucosal lesion (AGML). However, the patient was eventually diagnosed with phlegmonous gastritis because *Pseudomonas aeruginosa* and *S. viridans* were found in the culture of gastric juice.

Meanwhile, the culture of gastric tissue was negative for *Helicobacter pylori*. Antibiotic therapy (tazobactam/piperacillin, 9.0 g/day × 10 days) was then started, and gastric lesions gradually disappeared.

*H. pylori* were not identified using direct microscopy in all cases (Fig. 3b).

## Discussion

Phlegmonous gastritis is a rare inflammatory gastric disease caused by local or diffuse inflammation of the gastric wall [1]. The diffuse type is more common and has a higher mortality rate than the localized type. Case 1 was a diffuse type, whereas cases 2 and 3 showed a localized type. Imaging examinations are useful for diagnosing phlegmonous gastritis, which is suspected when gastric wall thickness is detected on abdominal ultrasonography or CT. Endoscopic ultrasonography for phlegmonous gastritis diagnosis is reportedly effective but is performed in few facilities [2].

By cause, phlegmonous gastritis is categorized into primary, secondary, and idiopathic types [3, 4]. The most common is the primary type, which is caused by a mucosal injury such as peptic ulcer or gastric cancer. The secondary type is associated with the infection of other organs, such as biliary infection or hepatic abscess. It may also occur after endoscopic submucosal dissection and endoscopic ultrasound-guided fine-needle aspiration [5, 6]. In the idiopathic type, as the name implies, the cause is unknown, and it mostly occurs in a compromised host. Moreover, cases 1 and 2 had a history of excessive alcohol consumption. Such a history is a causative factor of superficial gastritis, multiple small ulcers, and hemorrhagic erosions because alcohol can cause gastric mucosal injury, which induces phlegmonous gastritis [7]. Case 3 had no apparent background. However, he had an upcoming examination for promotion and felt extremely stressed. Mucosal injury such as AGML caused by mental stress could be the cause of phlegmonous gastritis [8]. Furthermore, temporary immunosuppression due to disruption in the rhythm of life might have triggered the onset of the disease in these three cases.

By clinical course, phlegmonous gastritis is categorized into acute, chronic, and subacute types [9]. All of our patients had a sudden onset of the disease, complaining of epigastralgia upon consultation. Therefore, they were categorized as the acute type.

The most common pathogenic cause of phlegmonous gastritis is *Streptococcus* (approximately 70%), which is resistant to gastric acid, followed by *Enterococcus* and *Staphylococcus* [10].

If the pathogen is found in the culture of gastric tissue or fluid, the diagnosis of phlegmonous gastritis could be made. However, pathogen is not always detected.

In case 1, although a pathogen was not identified, the patient was still diagnosed with phlegmonous gastritis because of other factors, such as the clinical course, symptoms, and imaging examinations.

Typically, the endoscopic image features of phlegmonous gastritis include reddish and edematous mucosa, swollen gastric folds, erosion, ulcer with slough, and mucopus adhesion. Considering that these features are nonspecific, various endoscopic images can be found in phlegmonous gastritis. Hence, we need to consider the differential diagnosis of phlegmonous gastritis.

*Case 1* Scirrhous gastric cancer was suspected according to the following endoscopic image features: extensively reddish and edematous gastric mucosa, swollen gastric folds, abundant mucopus adhesion, and poor gastric wall distensibility.

Histopathologically, no malignancy was noted.

Obtaining a clinical history for several months is important because scirrhous gastric cancer takes a long time course.

Ultimately, imaging and histopathological examinations led to the diagnosis of phlegmonous gastritis.

*Case 2* Gastric syphilis was suspected according to an endoscopic image feature, that is, multiple shallow ulcers with slough fused into a map-like pattern in the antrum. Furthermore, the patient was sexually active toward many unknown individuals. Regarding syphilis, the serum test and the immunostaining of the biopsy specimen were both negative (*Treponema pallidum* was not found). However, *S. viridans* was detected in the culture; thus, the patient was diagnosed with phlegmonous gastritis.

When gastric syphilis is suspected, serum test and skin examination must be conducted.

*Case 3* AGML was suspected because of the following endoscopic images: large and shallow ulcers with reddish and edematous mucosa and some signs of coagulation.

*P. aeruginosa* and *S. viridans* were found in the culture of gastric juice.

AGML is frequently caused by various factors, such as drugs (for example antibiotics, steroids, and nonsteroidal antiinflammatory agents), extreme mental or physical stress, and excessive alcohol consumption. Acute *H. pylori* infection must also be considered [11]. The patient had no history drug and alcohol intake and was negative for *H. pylori* infection. Considering that he had a history of mental stress, AGML caused by this stress was considered to be the cause of phlegmonous gastritis.

## Conclusion

The treatment for phlegmonous gastritis is conservative treatment using antibiotics. However, surgical treatment is required in resistant or severe cases [12]. Given that fatal cases were occasionally reported, physicians need to make a correct diagnosis and start the appropriate treatment [13].

## Data Availability

There are no additional data available for this study.

## Abbreviations

WBC: White blood cell
CT: Computed tomography
AGML: Acute gastric mucosal lesion
